# Toosendanin, a novel potent vacuolar-type H^+^-translocating ATPase inhibitor, sensitizes cancer cells to chemotherapy by blocking protective autophagy

**DOI:** 10.7150/ijbs.71041

**Published:** 2022-03-28

**Authors:** Yu Dong, Guoyuan Zhu, Sheng-Fang Wang, Kristine A. Keon, John L. Rubinstein, Si-Xin Zeng, Shuang Zhang, Qiu-Ling Chen, Jing Fu, Min Li, Han-Ming Shen, Jin-Jian Lu, Xiu-Ping Chen, Jia-Hong Lu

**Affiliations:** 1 State Key Laboratory of Quality Research in Chinese Medicine, Institute of Chinese Medical Sciences, University of Macau, Macau SAR, China.; 2 Guangdong-Hong Kong-Macau Joint Lab on Chinese Medicine and Immune Disease Research, University of Macau, Macau SAR, China.; 3 State Key Laboratory of Quality Research in Chinese Medicine, Guangdong-Hong Kong-Macao Joint Laboratory of Respiratory Infectious Disease, Macau Institute for Applied Research in Medicine and Health, Macau University of Science and Technology, Macau SAR, China.; 4 Molecular Medicine Program, The Hospital for Sick Children, 686 Bay Street, Toronto, Ontario, Canada M5G0A4.; 5 Department of Medical Biophysics, The University of Toronto, 101 College Street Suite 15-701, Toronto, Ontario Canada M5G 1L7.; 6 Department of Biochemistry, The University of Toronto, 1 King's College Circle, Toronto Ontario, Canada M5S 1A8.; 7 Mr. & Mrs. Ko Chi-Ming Centre for Parkinson's Disease Research, School of Chinese Medicine, Hong Kong Baptist University, Hong Kong SAR, China.; 8 Faculty of Health Sciences, University of Macau, Macau, China.

**Keywords:** toosendanin, V-ATPase inhibitor, autophagy inhibitor, protective autophagy, anti-cancer effect

## Abstract

Macroautophagy/autophagy is the process of self-digestion through the lysosomes; it disassembles unnecessary or dysfunctional long-lived proteins and damaged organelles for the recycling of biomacromolecules. Unfortunately, cancer cells can hijack this mechanism to survive under metabolic stress or develop drug resistance during chemotherapy. Increasing evidence indicates that the combination of autophagy inhibition and chemotherapy is a promising cancer treatment strategy. However, effective autophagy inhibitors with satisfied potency, bioavailability, and clearly-defined drug targets are still rare. Here, we report the identification of a potent autophagy inhibitor toosendanin which can effectively block autophagosome maturation, causing the accumulation of autophagy substrates in multiple cancer cells. Toosendanin did not inhibit the fusion process between autophagosome and lysosome but elevated lysosomal pH and impaired lysosomal enzymes activity. Using rat liver lysosome fraction and purified yeast V-ATPase, we found that toosendanin directly inhibited V-ATPase activity. By applying cellular thermal shift assay (CETSA), immunoprecipitation-coupled LC-MS/MS analysis, and biotin-toosendanin pull-down assay, we confirmed the direct binding between toosendanin and V-ATPase. Furthermore, toosendanin blocked chemotherapy-induced protective autophagy in cultured cancer cells and xenograft tumor tissues to significantly enhance anti-cancer activity. These results suggest that toosendanin has the potential to be developed into an anti-cancer drug by blocking chemotherapy-induced protective autophagy.

## Introduction

Cancers are a large group of diseases characterized by cellular genetic and metabolic changes that lead to uncontrolled cell growth with the potential to invade or spread to other parts of the body 1. Among biological mechanisms of cancer, increasing evidence indicates that dysregulation of autophagy is implicated in cancer initiation and progression. In general, autophagy prevents tumorigenesis by eliminating oncogenic stresses and maintaining genomic stability 2, 3. However, once the tumor is established, autophagy will become a surviving mechanism for cancer cells to adapt to the harsh microenvironment, including metabolic stress, hypoxia, or cell death induced by chemotherapy medications. In this case, autophagy empowers cancer cells with the potential invasion and metastasis capabilities and promotes tumor progression 4.

In recent years, increasing pharmacological studies have applied autophagy inhibitors for cancer treatment, and most of them indicate that autophagy inhibition can sensitize cancer cells to chemotherapy-induced cell death 5-12. Also, more than 35 clinical trials have been registered on the ClinicalTrials.gov website that focused on hydroxychloroquine or chloroquine (two FDA-approved anti-malaria drugs) as autophagy inhibitors in combination with chemotherapy drugs to treat various types of cancer. This increasing evidence suggests that the combination of autophagy inhibitor and chemotherapy medication is a promising cancer therapy strategy, and the enthusiasm for the discovery of small-molecule autophagy inhibitors for cancer therapy is still rising.

Toosendanin (TSN) is isolated from *Melia toosendan* Sieb. et Zucc which has been used to treat abdominal pain and used as the digestive tract parasiticide in ancient China for about 1500 years. Several studies have indicated that TSN shows potential anti-proliferative and pro-apoptotic effects on various human cancers 13-17, while the anti-cancer mechanism and drug target have not been fully understood. In this study, we identified a novel pharmacological effect of TSN: inhibition of V-ATPase activity to block late-stage autophagy. Furthermore, we studied the complementary effect of TSN on the anti-cancer efficacy of chemotherapy. Results showed that TSN inhibits camptothecin (CPT)-induced protective autophagy and significantly sensitizes cancer cells to CPT-induced toxicity in cellular and animal models, with good tolerance and safety.

## Results

### TSN inhibits autophagic flux

**Figure 1:**
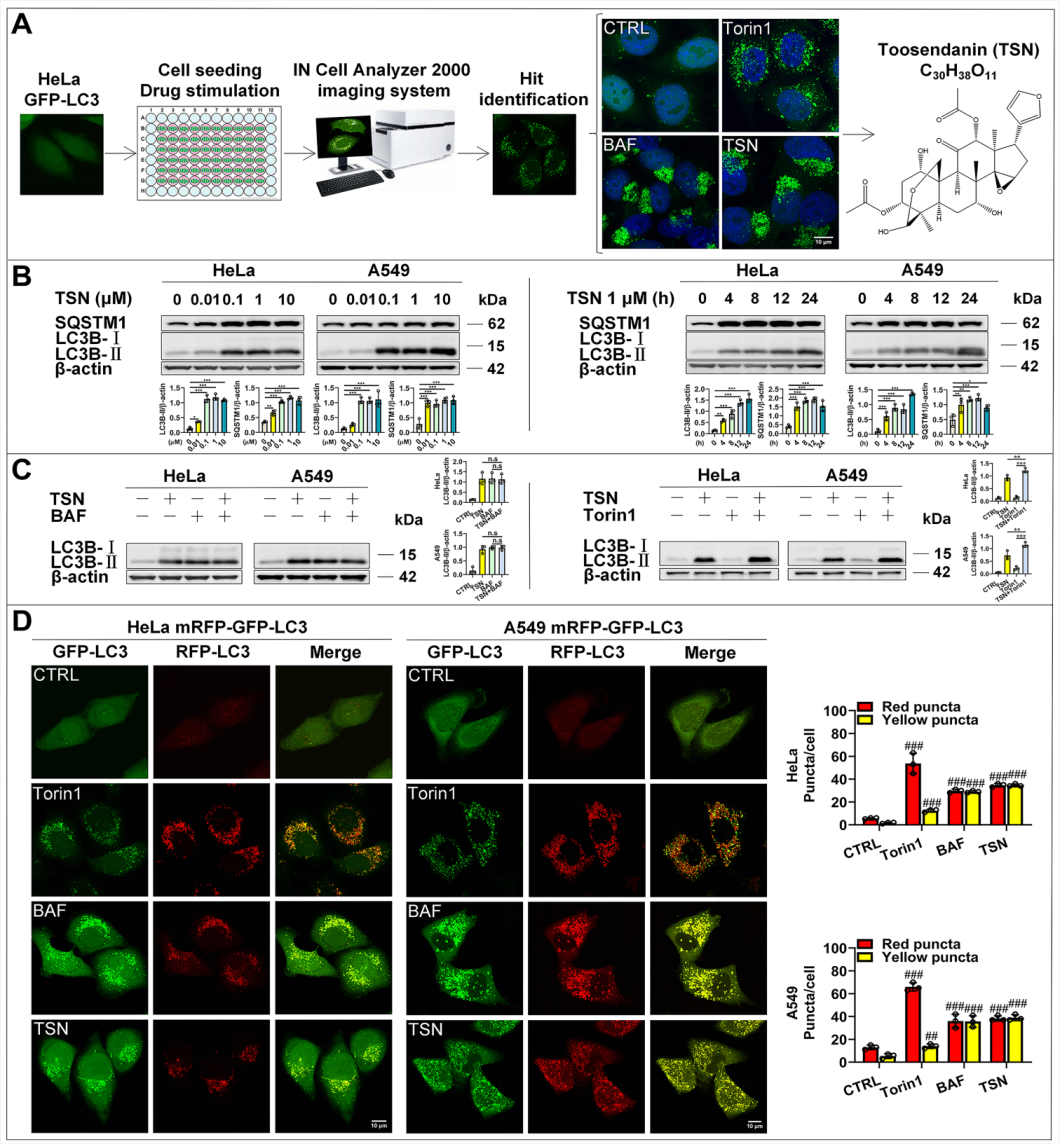
** The identification of the autophagy inhibitor TSN.** (A) Results of high-content screening for chemical autophagy modulators. (B) WB analysis of LC3B-II and SQSTM1/p62 protein levels in HeLa and A549 cells treated with TSN (0.01~10 μM) or TSN 1 μM for (0~24 h) (*n*=3). (C) WB analysis of LC3B-II protein level in HeLa and A549 cells treated with TSN 1 μM in the absence or presence of BAF 100 nM or Torin1 200 nM for 24 h (*n*=3). (D) HeLa and A549 cells stably expressing mRFP-GFP-LC3 were treated with Torin 200 nM, BAF 100 nM, TSN 1 μM for 12 h, and LSCM was applied to capture the fluorescent images (scale bar = 10 μm). The average number of red and yellow dots per cell was quantified (*n*≥30, 3 biological repeats). ^##^*P*<0.01, ^###^*P*<0.001 (*vs*. Control), ^*^P<0.05, ^**^P<0.01. ^***^P<0.001. ns, not significant.

To search for small molecules that block autophagic flux, we applied the high-content screening to observe the effect of compounds on the distribution of GFP-LC3 puncta in HeLa cell line with stable expression of GFP-LC3. GFP-LC3 is evenly distributed in the cytoplasm under normal conditions but dramatically accumulates to form puncta under either autophagy induction or autophagy maturation inhibition conditions. TSN was identified as the autophagy modulator by IN CELL Analyzer 2000 from our natural compound library (>3000 compounds) and further confirmed by laser scanning confocal microscopy (Figure 1A). TSN induced the dramatic accumulation of GFP-LC3 puncta in a pattern similar to that induced by autophagy inhibitor bafilomycin A_1_ (BAF), and was thus identified as an autophagy modulator.

To further determine whether TSN-induced accumulation of GFP-LC3 puncta is due to increased autophagosome biogenesis or blocked autophagosome maturation/degradation, we followed the Autophagy Modulator Scoring System (AMSS) and completed a series of assays 18. First, we observed TSN-induced autophagosome accumulation through GFP-LC3 puncta (Figure 1A). Next, we tested autophagosome formation-related biochemical change and found that TSN significantly increased LC3-II level in both dose-dependent and time-dependent manners in HeLa and A549 cells (Figure 1B). Since either autophagosome biogenesis or the blockage of autophagosome maturation can cause the increased LC3-Ⅱ level and autophagosome number, we then monitored the autophagic flux using the LC3-II turnover assay (Figure 1C) and mRFP-GFP tandem fluorescent-tagged LC3 (mRFP-GFP-LC3) (Figure 1D) 19, 20. First, TSN could not further increase the LC3-II level in the presence of the lysosomal inhibitor BAF, but could further increase the LC3-II level in the presence of the autophagy inducer Torin1 in HeLa and A549 cells. Second, we found a few GFP and RFP puncta accumulated in the control group, which reflected the basal autophagic flux in HeLa and A549 cells. However, under autophagy induction (Torin1-treated group) 21, GFP and RFP puncta significantly increased and accumulated on the autophagosome membrane, but GFP puncta were quenched within the acidic autolysosomes, leaving more RFP puncta to be observed because RFP is more resistant to the acidic environment. When the autophagosome-lysosome fusion process or lysosomal function is impaired (BAF treatment) 22, GFP puncta will not be quenched, thus yielding more yellow puncta (GFP- and RFP- positive autophagosomes or autolysosomes) to be observed. After TSN treatment, we found a similar pattern as the BAF-treated cells, suggesting that TSN also inhibited the autophagic flux. Finally, we found that TSN significantly increased the autophagy substrate SQSTM1/p62 protein level (Figure 1B). Collectively, these data suggested that TSN is capable of blocking the late-stage autophagy.

### TSN impairs the lysosomal acidic environment and cathepsins maturation

**Figure 2:**
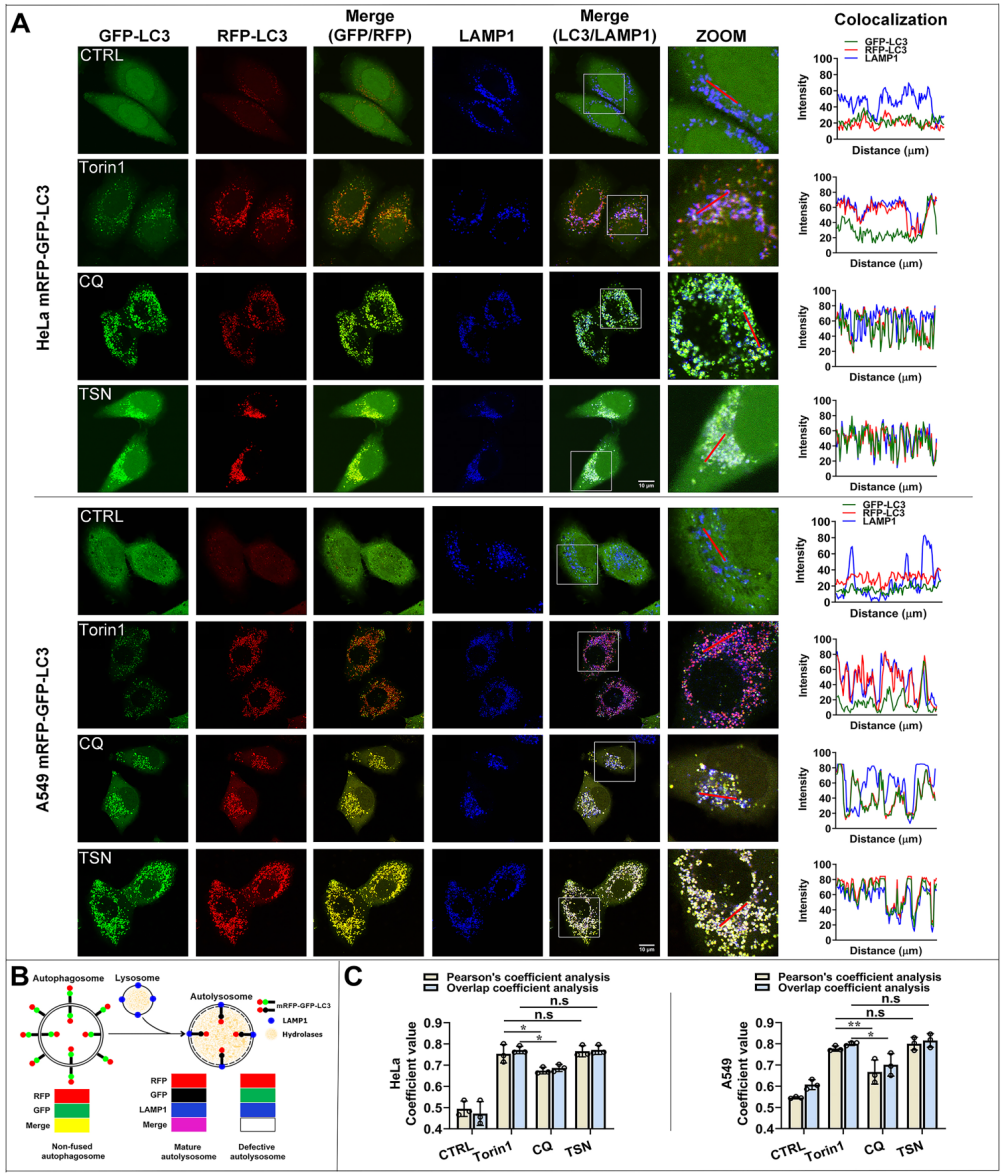
** TSN does not inhibit the autophagosome-lysosome fusion proces**s. (A) HeLa and A549 cells stably expressing mRFP-GFP-LC3 were treated with Torin1 200 nM, CQ 30 μM, TSN 1 μM for 12 h, followed by immunostaining of LAMP1, and LSCM was applied to capture the fluorescent images (scale bar = 10 μm). The colocalization of GFP-, RFP- and LAMP1- puncta in cells was analyzed (*n*≥30, 3 biological repeats). (B) The illustration of the non-fused autophagosome, defective autolysosome, and mature autolysosome analyzed by the colocalization of GFP-, RFP-, and LAMP1- puncta. (C) Colocalization analysis between RFP- and LAMP1- puncta, Pearson's coefficient and overlap coefficient values were calculated by ImageJ (JACoP, Just Another Colocalization Plugin) (*n*≥10, 3 biological repeats). ^*^P<0.05, ^**^P<0.01. ns, not significant.

Both defective autophagosome-lysosome fusion process and dysfunctional lysosomes can cause the blockage of autophagosome maturation and degradation 23, 24. We first checked whether TSN inhibited the autophagosome-lysosome fusion process; we performed qualitative and quantitative colocalization analyses of mRFP-GFP-LC3 and LAMP1 puncta (Figure 2A-C). In torin1-treated cells, autophagosomes fused with lysosomes, and then GFP puncta were quenched in the acidic autolysosomes, leaving pinker puncta (the colocalization of RFP- and LAMP1- puncta). In CQ-treated cells, we observed that part of yellow puncta (the colocalization of RFP- and GFP- puncta) could not colocalize with blue puncta (LAMP1-puncta), suggesting partial autophagosomes could not fuse with lysosomes. However, in TSN-treated cells, we observed more white puncta (the colocalization of GFP-, RFP- and LAMP1- puncta), suggesting the autophagosomes could fuse with lysosomes.

Besides, Pearson's coefficient value and overlap coefficient value are the representative parameters for colocalization studies and the value one stands for a complete colocalization situation 25. Torin1 did not affect the fusion process. After CQ treatment, Pearson's coefficient value and overlap coefficient value were slightly but significantly lower than torin1 treatment, suggesting that CQ partially blocked the autophagosome-lysosome fusion process. After TSN treatment, Pearson's coefficient and overlap coefficient values were similar to the torin1 treatment and showed no significant difference, suggesting that TSN did not affect the autophagosome-lysosome fusion process.

**Figure 3:**
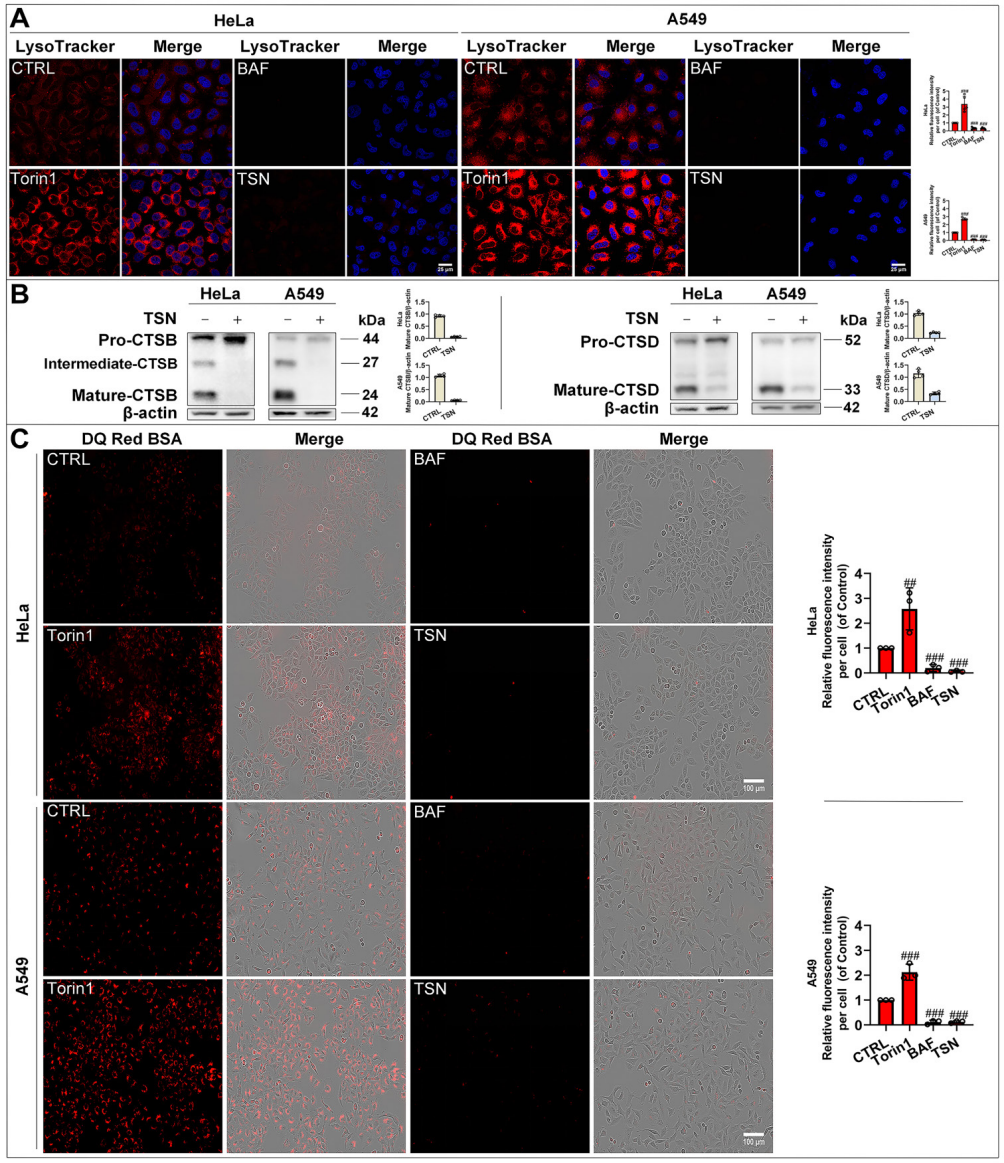
** TSN impairs lysosomal proteases hydrolytic activity.** (A) HeLa and A549 cells were treated with Torin1 200 nM, BAF 100 nM, TSN 1 μM for 24 h, followed by LysoTracker Red staining, and LSCM was applied to capture the fluorescent images (scale bar = 25 μm). The cell nuclei were stained with Hoechst. The relative red fluorescence intensity per cell in each treatment was measured and analyzed (*n*≥30, 3 biological repeats). (B) WB analysis of CTSB and CTSD protein levels in HeLa and A549 cells treated with 0.1% DMSO or TSN 1 μM for 24 h (*n*=4). (C) HeLa and A549 cells were pre-incubated with DQ Red BSA for 2 h, and then treated with Torin1, BAF, or TSN 400 nM for 6 h, and IncuCyte S3 live-cell analysis system was applied (scale bar = 100 μm). The relative red fluorescence intensity per cell in each treatment was measured and analyzed (*n*≥30, 3 biological repeats). ^##^*P*<0.01, ^###^*P*<0.001 (*vs*. Control).

To confirm whether TSN impairs the lysosomal acidic environment, we used the LysoTracker Red dye, which labeled acidic organelles, e.g., lysosomes (Figure 3A). Torin1 induced the translocation of transcription factor EB (TFEB) into the nucleus and upregulated the autophagy- and lysosome biogenesis-related gene expression 26, 27. Therefore, we observed an increase in red fluorescence intensity in Torin1 treated-cells. On the contrary, V-ATPase inhibitor BAF impaired the lysosomal acidic environment 28. Thus, we found there was a significant decrease in red fluorescence intensity in BAF treated-cells. Similarly, TSN treatment group also showed a dramatic decrease in red fluorescence intensity. Besides, the lysosomal acidic environment is essential for the activation of cathepsins and the degradation of their substrates through the autophagosome-lysosome pathway 29. Cathepsin B (CTSB) and Cathepsin D (CTSD) are abundant lysosomal proteases. First, we found that mature-CTSB and mature-CTSD protein levels were significantly decreased in TSN-treated cells (Figure 3B), suggesting that TSN impaired the lysosomal acidic environment and inhibited the cathepsins maturation process. Second, we applied the molecular probe DQ Red BSA to monitor mature cathepsins' hydrolytic activity (Figure 3C). Within the lysosomal acidic environment, DQ Red BSA is cleaved by proteolytic enzymes and releases red fluorescent peptide fragments 30. In the control group, we observed the red fluorescence within the cell, indicating the undergoing basal hydrolytic activity of mature cathepsins. Next, we observed an increase in red fluorescence intensity in Torin1 treated-cells, indicating the upregulated lysosomal hydrolytic activity. Meanwhile, showing a similar effect to BAF treatment group, there was a significant decrease in red fluorescence intensity in TSN-treated cells, suggesting that TSN impaired the lysosomal proteolytic activity. Besides, we also found that TSN did not decrease the protein level of lysosomal-associated membrane protein 1 (LAMP1), and the morphology of lysosomes was also similar to those in the control group, indicating that TSN might not inhibit the late-stage autophagy by directly destroying the integrity of the lysosome or reducing lysosome numbers (Figure 2A, S1A).

### TSN physically binds to the V-ATPase and inhibits its proton pumping ability

Lysosomal ion channels and transporters play essential roles in maintaining lysosomal homeostasis, and as an ATP-dependent proton pump, V-ATPase establishes and maintains the lysosomal environment while yielding a highly acidic pH for the activation of hydrolytic enzymes 31, 32. Also, pharmacological studies indicate that small molecules can impair the lysosomal acidic environment via inhibiting the V-ATPase activity 33. To confirm whether TSN inhibits the V-ATPase activity, we performed the conventional ATP/NADH (NADPH)-coupled assay 34, 35. An ATP molecule is hydrolyzed, and an NADH (NADPH) molecule is simultaneously oxidized. Because NADH (NADPH) absorbs light strongly at 340 nm, we can detect the rate of ATP hydrolysis and V-ATPase activity by measuring the decrease in optical density (OD) value by a spectrophotometer.

**Figure 4:**
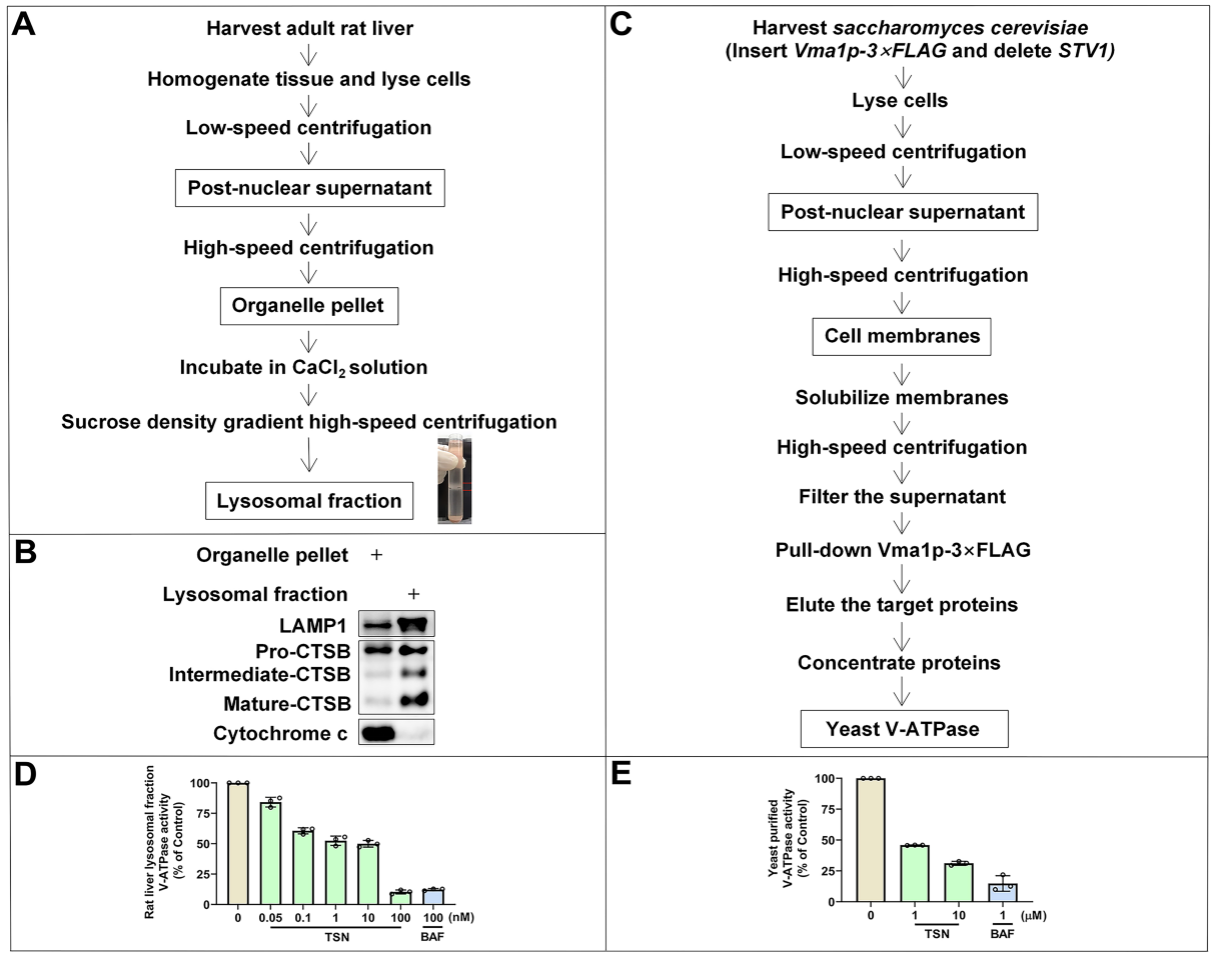
** TSN inhibits the V-ATPase activity.** (A) The isolation process of rat liver lysosomal fraction. (B) WB analysis of LAMP1, CTSB and cytochrome C protein levels in the organelle pellet and lysosomal fraction. (C) The isolation process of yeast V-ATPase. (D)-(E) Rat liver lysosomal and purified yeast V-ATPase activity assay in TSN and BAF treatment groups (*n*=3).

We first isolated the lysosomal fraction from the rat liver with the sucrose density gradient centrifugation (Figure 4A) and detected the lysosomal biomarkers (LAMP1 and CTSB) and mitochondrial biomarker (cytochrome C) to confirm the enrichment of lysosomal fraction (Figure 4B). BAF is the classical V-ATPase inhibitor and was used as the positive control group. Showing a similar effect to BAF, TSN also inhibited the activity of V-ATPase in rat liver lysosomes in a dose-dependent manner (Figure 4D). Next, we used purified yeast V-ATPase to confirm the inhibitory effect of TSN on different organisms. Yeast Vma1p (homo sapiens V_1_A homolog) is one of the largest subunits in the V_1_ complex; we purified intact yeast V-ATPase from detergent-solubilized membranes using a strain with the sequence encoding a *3×FLAG* tag inserted into its chromosomal DNA 3′ of the gene encoding *Vma1p (A)* (Figure 4C). We confirmed that TSN inhibited the activity of purified yeast V-ATPase (Figure 4E, S1B).

**Figure 5:**
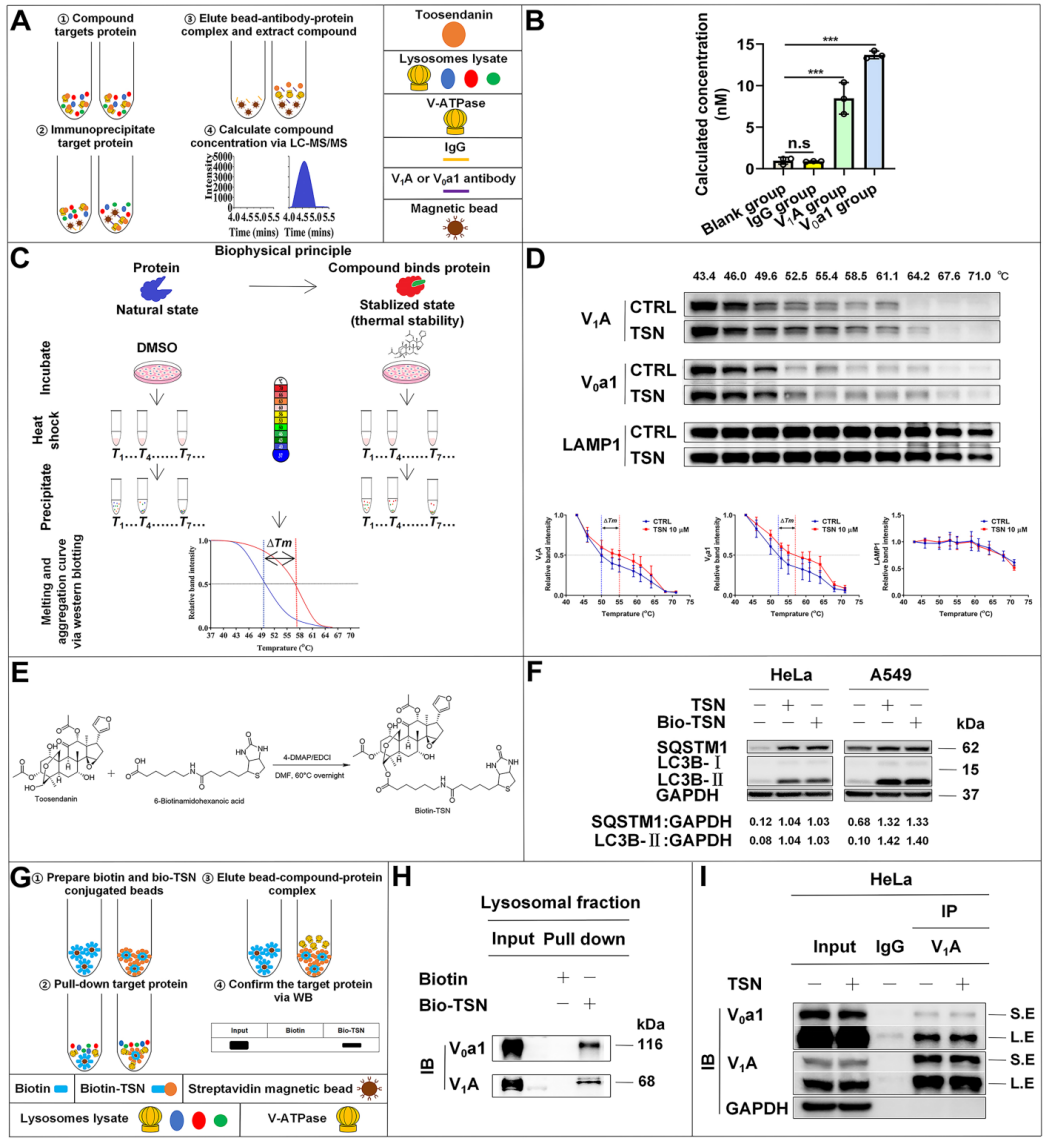
** TSN directly binds to V-ATPase.** (A)-(B) Immunoprecipitation-coupled LC-MS/MS analysis. Lysosomes lysate was incubated with TSN for 2 h, and then subject to immunoprecipitation with IgG, V_1_A, and V_0_a1 antibodies. The proteins were precipitated by methanol and the concentration of TSN was analyzed by LC-MS/MS analysis (*n*=3). (C)-(D) Cellular thermal shift assay. HeLa cells were treated with 0.1% DMSO or TSN for 1 h, and followed by 3 min heating at indicated temperature (43 to 71 ℃). The cell lysates were analyzed by WB to determine the V_1_A and V_0_a1 protein levels. The band intensity in each temperature was normalized to the band intensity at the lowest temperature and the thermal aggregation curves is made following the quantification of the WB (*n*=3). (E) Biotin-TSN synthesis process. (F) WB analysis of LC3B-II and SQSTM1/p62 protein levels in HeLa and A549 cells treated with TSN or bio-TSN 1 μM for 24 h. The ratio of target protein/GAPDH was calculated based on the band intensity. (G)-(H) Bio-TSN pull-down assay. Biotin or Bio-TSN conjugated streptavidin magnetic beads were incubated with lysosomal fraction to pull down the Biotin and Bio-TSN binding proteins, and the presence of V-ATPase in the purification fraction was determined by WB detection of V_0_a1 and V_1_A. (I) Co-immunoprecipitation assay. HeLa cells were treated with 0.1% DMSO or TSN 100 nM for 12 h, followed by co-immunoprecipitation of IgG and V_1_A, detected the protein levels of V_0_a1, V_1_A, and GAPDH. S.E, short-time exposure; L.E, long-time exposure. ^***^P<0.001. ns, not significant.

Next, to understand how TSN inhibits V-ATPase activity, we performed a series of biochemical analyses to understand whether TSN directly binds to the V-ATPase and inhibits its proton pumping ability. Firstly, we have developed a novel label-free method titled immunoprecipitation-coupled LC-MS/MS analysis for the detection of compound-protein physical binding. We incubated the lysosomal fractions with TSN and pulled down the V-ATPase complex by two individual subunits antibodies, then extracted the compound and performed LC-MS/MS to confirm whether TSN could bind to V-ATPase. As expected, we found that V_1_A and V_0_a1 antibodies, but not IgG control, could significantly pull down TSN (Figure 5A-B).

Then, we applied the cellular thermal shift assay (CETSA) 36 to confirm the physical interaction between TSN and V-ATPase. Theoretically, the direct binding of the drug to the target protein modulates the conformation and thermal stability of protein; thus, the protein complex will exhibit resistance to thermal denaturation (Figure 5C). Thermal profiling of V_1_A and V_0_a1 (two large subunits of V-ATPase) with TSN treatment yielded a sizable stabilization with an overall thermal shift (∆*Tm*, the melting temperature) of up to about 5 ℃, indicating TSN interacted with the V-ATPase and improved its resistance to thermal denaturation (Figure 5D). LAMP1 is a lysosomal-associated membrane protein; thermal profiling of LAMP1 was performed here as a negative control group, and we found that TSN treatment could not protect LAMP1 from thermal denaturation (Figure 5D).

Next, we took advantage of the high binding affinity between biotin and streptavidin and synthesized the biotin-conjugated TSN (bio-TSN) (Figure 5E). Then, we performed NMR, WB, and immunofluorescence to confirm the structure and autophagy inhibition of bio-TSN (Figure 5F, S2A). Next, we incubated the lysosomal fractions with bio-TSN conjugated streptavidin magnetic beads, pulled down its target proteins, and then detected the protein levels of V_1_A and V_0_a1 by WB to confirm that bio-TSN interacted with V-ATPase (Figure 5G-H).

V_1_A and V_0_a1 are the two largest subunits in the V_1_ subcomplex and V_0_ subcomplex, respectively, and the V-ATPase activity can be regulated by the association and disassociation of these two subcomplexes 37. If TSN disrupts the interaction of two or more subunits among V-ATPase subunits, it will dissociate the physical interaction between V_1_A and V_0_a1. As seen in Figure 5I and S1C, we found that TSN did not impair the interaction between V_1_A and V_0_a1 and did not decrease these two subunits' protein levels, indicating that TSN does not destroy the intact structure of V-ATPase. Collectively, these data indicate that TSN physically binds to V-ATPase to interrupt its ATP hydrolysis and proton translocation activity.

### TSN sensitizes cancer cells to chemotherapy by blocking protective autophagy

As the traditional chemotherapy medications, camptothecin (CPT) and its derivatives are widely used for cancer therapy 38. However, the occurrence of drug resistance would limit their successful therapeutic effect 39. Among various factors, autophagy is considered a surviving mechanism for cancer cells during chemotherapy 40, 41. Our previous study found that CPT induced protective autophagy in multiple cancer cells to promote drug resistance 42. Here, we planned to test whether the small-molecule autophagy inhibitor TSN could enhance the cytotoxicity of CPT on cancer cells.

**Figure 6:**
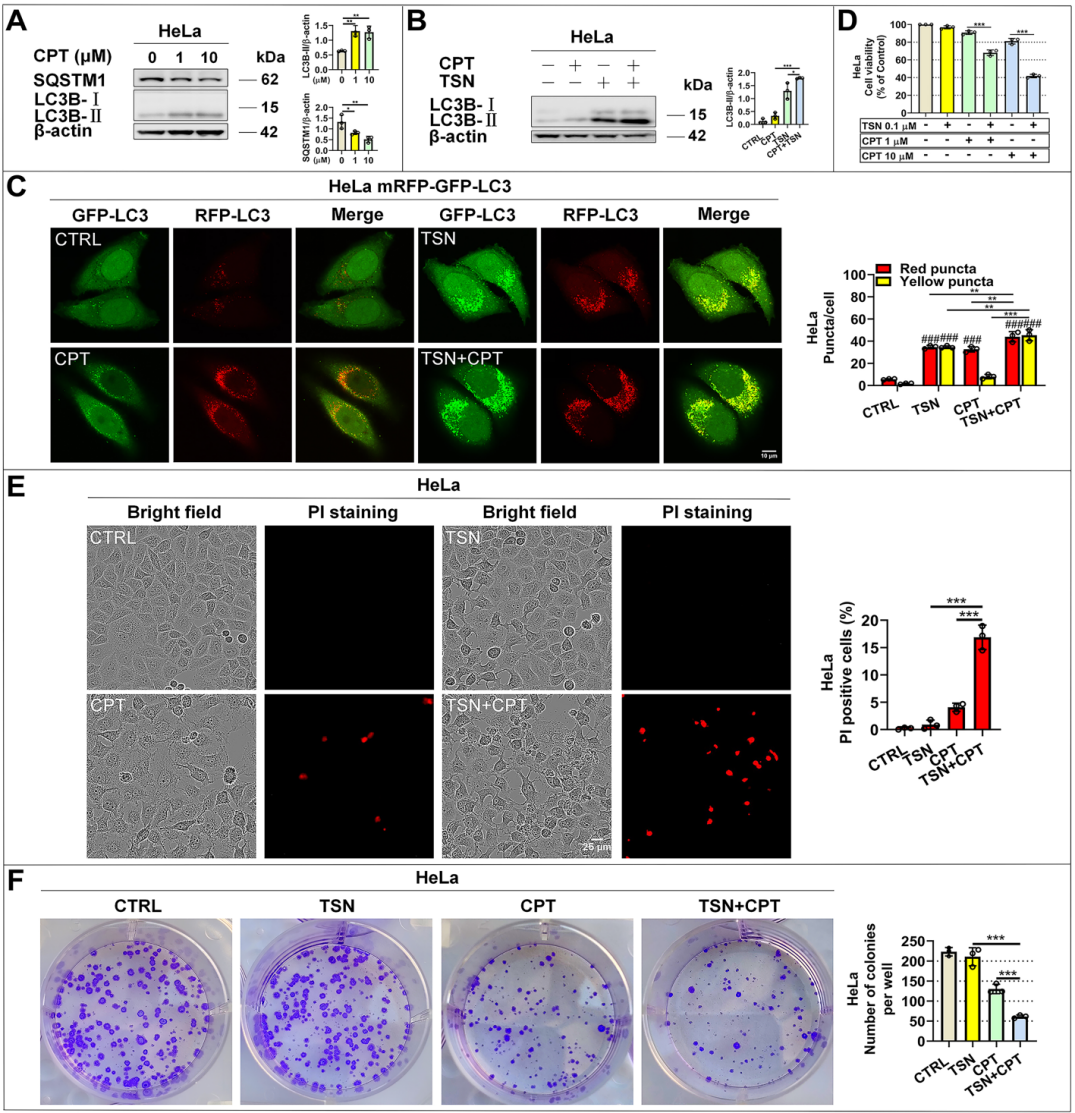
** TSN inhibits CPT-induced autophagy and enhances its anti-cancer activity *in vitro*.** (A) WB analysis of LC3B-II and SQSTM1/p62 protein levels in HeLa cells treated with CPT for 24 h (*n*=3). (B) WB analysis of LC3B-II protein level in HeLa cells treated with CPT 1 μM in the absence or presence of TSN 1 μM for 24 h (*n*=3). (C) HeLa cells stably expressing mRFP-GFP-LC3 were treated with CPT 1 μM in the absence or presence of TSN 1 μM for 12 h, and LSCM was applied to capture the fluorescent images (scale bar = 10 μm). The average number of red and yellow dots per cell was quantified (*n*≥30, 3 biological repeats). (D) HeLa cells were treated with TSN and CPT for 24 h, and LDH release assay was performed to assess the cell viability (*n*=3). (E) HeLa cells were treated with TSN 100 nM and CPT 10 μM for 24 h, followed by PI staining, and IncuCyte S3 live-cell analysis system was applied (scale bar = 25 μm). PI-positive cells in each group were quantified (*n*=3). (F) HeLa cells were treated with CPT 10 nM in the absence or presence of TSN 1 nM for 7~10 d; cells were stained with crystal violet. The colony numbers in each treatment were quantified (*n*=3). ^###^*P*<0.001 (*vs*. Control), ^*^P<0.05, ^**^P<0.01, ^***^P<0.001.

We first reconfirmed that CPT induced autophagy in HeLa cells as evidenced by increased LC3-Ⅱ and decreased SQSTM1/p62 protein levels in a dose-dependent manner (Figure 6A). Then, we found that CPT further increased LC3-Ⅱ level in the presence of TSN (Figure 6B), indicating the blockage of autophagic degradation of LC3-II. Also, CPT induced dramatic red-only puncta (mature autophagosome formation in HeLa cells stably expressing mRFP-GFP-LC3), while TSN co-treatment prevented the formation of red-only puncta and led to a significant increase of yellow puncta (immature autophagosome) (Figure 6C). We next obtained the cell viability of the two compounds (Figure S3A, B) and studied whether TSN could enhance the anti-cancer activity of CPT by blocking CPT-induced autophagy. As seen in Figure 6D, compared with CPT or TSN treatment alone, the co-treatment further significantly decreased the cell viability in a dose-dependent manner. Propidium iodide (PI) is membrane impermeable and generally excluded from viable cells; we used PI to identify dead cells in each treatment. The result indicated that the co-treatment significantly caused cell death compared with CPT or TSN treatment alone (Figure 6E). Also, colony formation assay showed that CPT further significantly inhibited cancer cell colony formation in the presence of TSN (Figure 6F, S3C). These data collectively suggested that TSN inhibited CPT-induced autophagy and enhanced its toxicity towards HeLa cells *in vitro*.

**Figure 7:**
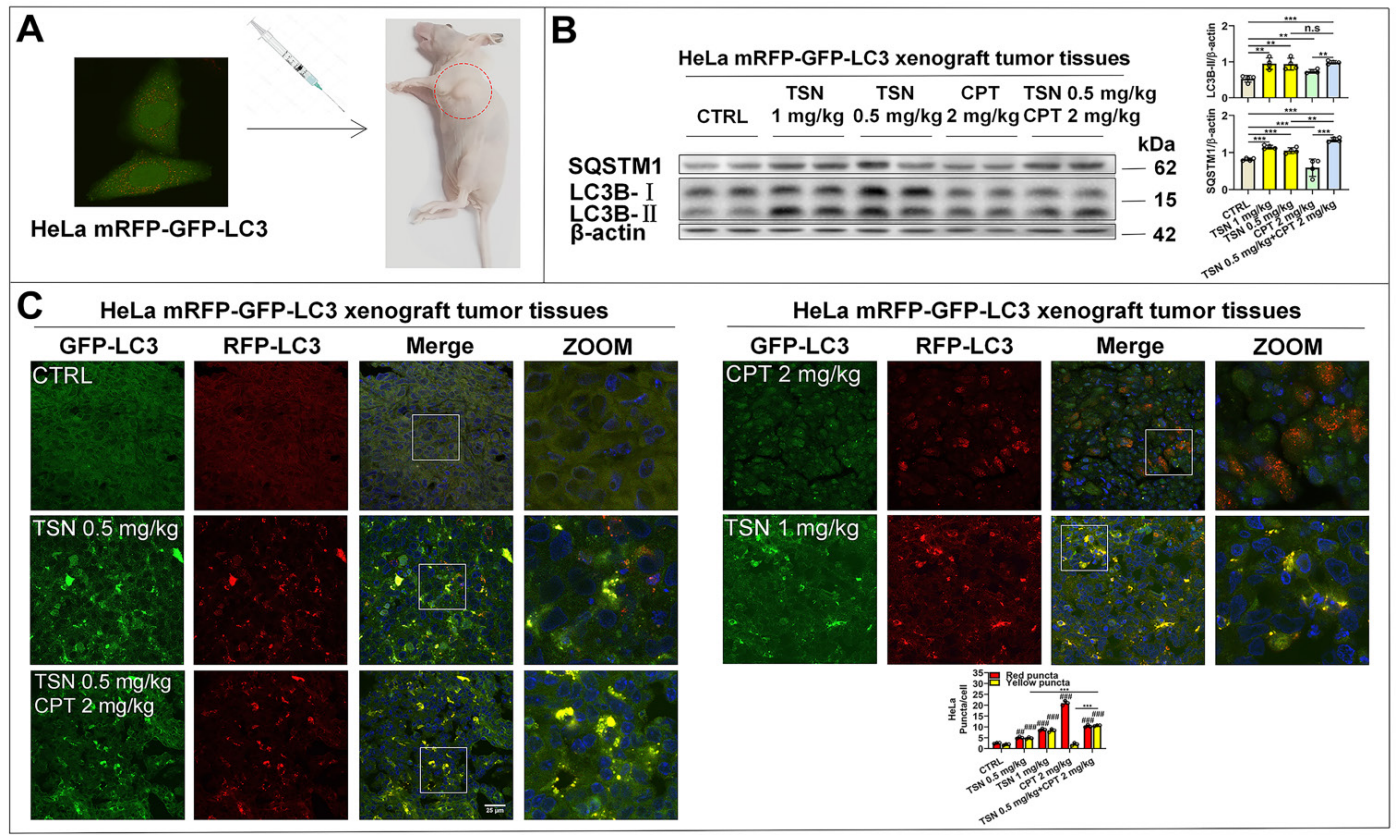
** TSN inhibits CPT-induced autophagy in HeLa cell line (stably expressing mRFP-GFP-LC3) xenograft models.** (A) The cervical cancer nude mice models were established. (B) WB analysis of LC3B-II and SQSTM1/p62 protein levels in the tumor tissues (*n*=4). (C) Frozen sections of tumor tissues were photographed by LSCM (scale bar = 25 μm). The cell nuclei were stained with Hoechst. The average number of red and yellow dots per cell was quantified in at least 30 cells from each animal. 3 animals in each group were analyzed. ^##^*P*<0.01, ^###^*P*<0.001 (*vs*. Control), ^**^P<0.01, ^***^P<0.001, ns, not significant.

Next, we established HeLa mRFP-GFP-LC3 xenograft nude mice models to visualize the autophagic flux in xenograft tumor tissues (Figure 7A). We referenced previous studies and determined the dosage of CPT and TSN in this study 43, 44. Once tumor grafts formed, the mice received intraperitoneal injection of vehicle, CPT (2 mg/kg), TSN (0.5 and 1 mg/kg), or CPT (2 mg/kg) + TSN (0.5 mg/kg) every 2 days for 3 weeks. We confirmed that administration of CPT to mice obviously induced autophagy in tumor tissues, as shown by increased LC3-II level and decreased SQSTM1/p62 level in tumor tissue lysates and massive formation of red-only puncta in tumor tissue sections (Figure 7B-C). Compared with CPT treatment alone, the co-treatment with TSN further increased both LC3-II and SQSTM1/p62 protein levels, blocked the formation of red-only puncta, and increased yellow puncta number in the tumor tissues. These data suggested that TSN effectively inhibited CPT-induced autophagy in tumor tissues.

**Figure 8:**
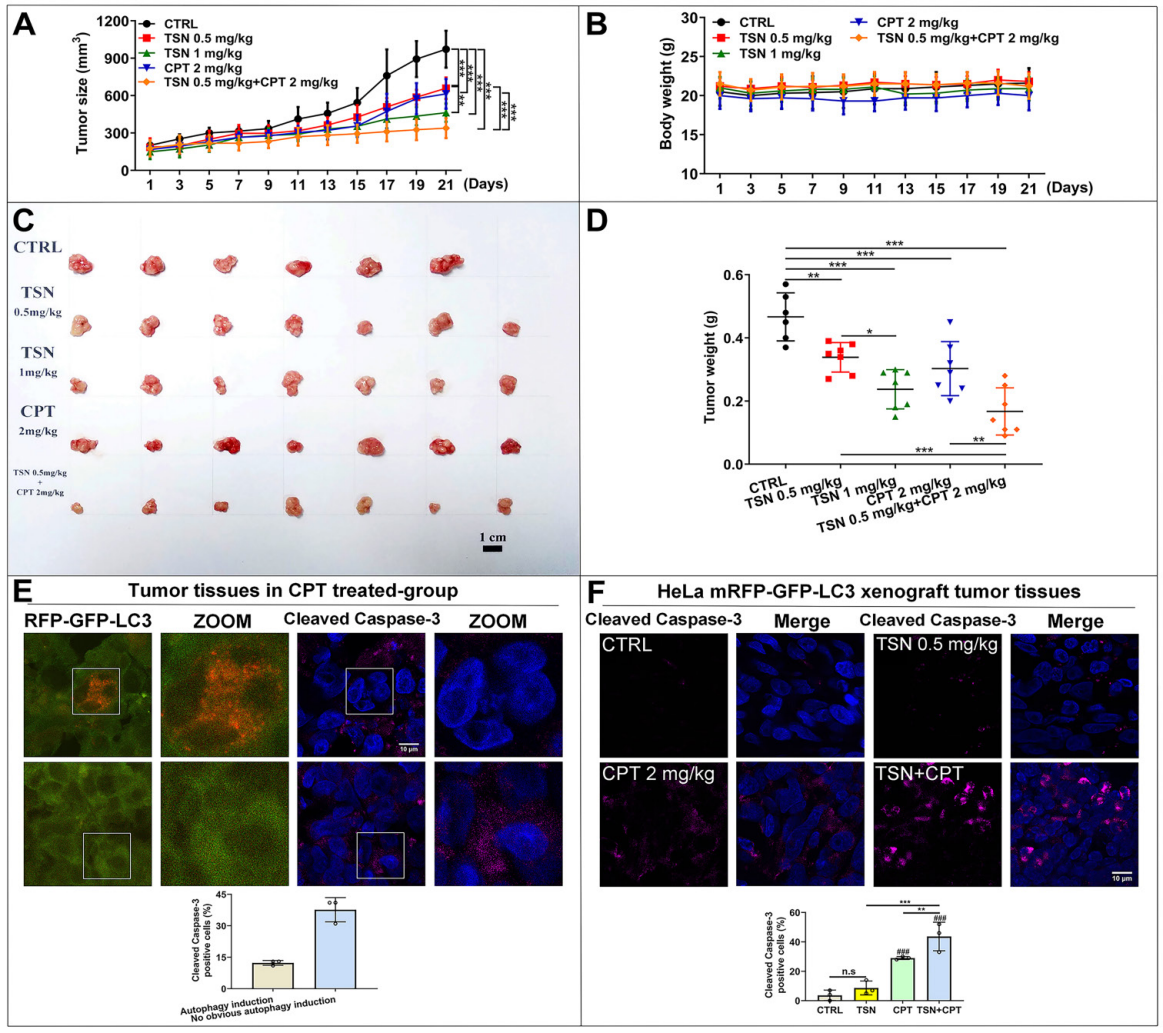
** TSN enhances the anti-cancer activity of CPT in HeLa cell line (stably expressing mRFP-GFP-LC3) xenograft models.** (A) Tumor size was measured every 2 days with a caliper, and the volume was calculated by the formula (length × width^2^)/2. (B) Body weight was measured every 2 days. (C) The tumors were isolated on the day of sacrifice; images showed the tumor morphology in each group (scale bar = 1 cm). (D) Tumor weight was measured on the day of sacrifice. (E) Representative images showed the immunofluorescence staining of cleaved caspase-3 in CPT-treated group, and LSCM was applied to capture the fluorescent images (scale bar = 10 μm). The cell nuclei were stained with Hoechst. Cleaved caspase-3 positive cells were quantified according to autophagic flux (*n*≥30 cells, 3 animals). (F) Representative images showed the immunofluorescence staining of cleaved caspase-3 in each group, and LSCM was applied to capture the fluorescent images (scale bar = 10 μm). The cell nuclei were stained with Hoechst. Cleaved caspase-3 positive cells in each group were quantified in at least 30 cells from each animal. 3 animals in each group were analyzed. ^###^*P*<0.001 (*vs*. control), ^**^P<0.01, ^***^P<0.001. ns, not significant.

As shown in Figure 8A, tumor size increased dramatically in the control group but was significantly suppressed in the other four treatments. The co-treatment group (TSN 0.5 mg/kg plus CPT 2mg/kg) exhibited the highest tumor growth inhibition effect, followed by TSN (1 mg/kg), CPT (2 mg/kg) and TSN (0.5 mg/kg) (Figure 8A, C, D). Moreover, there was no significant difference in the mean body weight among the five groups (Figure 8B), and histological analysis showed that administration of TSN and CPT did not cause the obvious damage and morphology change of the livers, kidneys, and spleens (Figure S4A), indicating that the nude mice well tolerated all treatments with no obvious toxicity during the whole experiment. Caspase-3 is a critical executioner of apoptosis since its activation form is responsible for the proteolytic cleavage of many key proteins that promote apoptosis cascade 45. Therefore, we stained endogenous cleaved caspase-3 to confirm whether a) CPT could induce the protective autophagy in the tumor tissues, b) TSN could enhance the anti-cancer effect of CPT* in vivo*. Interestingly, we found cleaved caspase-3 signals decreased in autophagy induction cells (more RFP puncta cells) after CPT treatment but increased in the cells with no obvious autophagy induction (Figure 8E). It indicated that CPT might promote cell survival via the up-regulation of autophagy, i.e., protective autophagy. We then quantified cleaved caspase-3 positive cells in each treatment, and as seen in Figure 8F, compared with CPT or TSN treatment alone, the co-treatment further significantly increased the cleaved caspase-3 positive cells, suggesting that the co-treatment further caused cell apoptosis. Collectively, the data indicate that TSN enhanced the therapeutic efficacy of CPT in HeLa mRFP-GFP-LC3 xenograft nude mice models by inhibiting the protective autophagy.

## Discussion

Chemotherapy, radiotherapy, and targeted therapy are essential anti-cancer treatments to suppress cancer growth, prevent cancer relapse, and prolong patients' survival. However, the emergence of drug resistance in anti-cancer therapies has become a major obstacle to the success of cancer therapy 46, 47. It is worth noting that autophagy induction has been recognized as an important mechanism for tumor cells to develop drug resistance, and autophagy inhibition has been shown to evade the drug resistance and further enhance the cytotoxicity of anti-cancer therapies 48. Although an increased number of autophagy inhibitors have been identified, the lack of potent efficacy with a clearly defined drug target is still the main obstacle to the development of autophagy modulators in clinical applications 49, 50.

Natural products provide a rich and valuable source for the discovery of autophagy modulators, such as rapamycin, resveratrol, trehalose, rottlerin, quercetin, and sulforaphane 51-56. However, small-molecule compounds isolated from natural products usually exhibit moderate autophagy modulation. TSN was mainly extracted from *Melia toosendan* Sieb. et Zucc., and showed anti-parasitic in the traditional application. In this study, we identified TSN as an autophagy modulator through a high-content screening model and further confirmed it blocks the autophagic flux and inhibits its substrates degradation. Interestingly, TSN showed highly potent autophagy inhibition activity in both cancer cell lines and tumor xenograft nude mice models. We found that TSN can efficiently inhibit autophagic flux at a relative low concentration (10 nM in cell culture and 0.5 mg/kg (i.p.) in mice), indicating that TSN is a potent autophagy inhibitor.

Drug target identification is a challenging task. V-ATPase contains a peripheral V_1_ domain and an integral membrane V_0_ domain. Each domain comprises multiple subunits, which tethers together for ATP hydrolysis and proton translocation 37, 57. Therefore, it might not be easy to confirm the direct interaction between the TSN and the large V-ATPase complex through the technology of surface plasmon resonance (SPR) or isothermal titration microcalorimetry (ITC). Under this consideration, we developed and applied biochemical analyses to confirm that V-ATPase complex can pull down TSN and biotin-TSN can also pull down V-ATPase complex, indicating that TSN physically binds and inhibits V-ATPase activity. In this study, we used the NP40 lysis buffer in the identification of drug target, because SDS lysis buffer will disrupt the interaction between TSN and V-ATPase. Therefore, further analysis such as crystal structure may help clarify the inhibitory mechanism of TSN on a specific subunit of V-ATPase. In recent years, increasing evidence has shown that V-ATPase is involved in many important physiological processes, including autophagy, endocytosis, immunomodulation, extracellular acidification, Warburg effect, Notch, Wnt, TGF-β, and mTOR signaling pathways. Therefore, as a potent V-ATPase inhibitor, TSN may also be a promising drug candidate for the treatment of diseases with abnormally elevated cell acidity, including autoimmune diseases, osteoporosis, and cancers 58.

Our research focuses on autophagy-induced tumor tolerance and examines whether autophagy inhibition can enhance anti-cancer therapy by overcoming drug resistance. Due to the challenge of monitoring autophagy flux in animal tumor tissues, we established the HeLa mRFP-GFP-LC3 xenograft nude mice models and confirmed that it can accurately reflect the induction or inhibition of autophagy. Also, these tumor-bearing mice can well characterize protective autophagy in tumor tissues treated with chemotherapeutic drugs. Our results confirmed that apoptotic cells decreased in autophagy-inducing tumor tissues, indicating that the tumor hijacks the autophagy-lysosomal degradation pathway to resist chemotherapy-induced cell death. Although we found that CPT and TSN treatment alone showed anti-cancer activity, CPT and TSN co-treatment can further significantly induce cancer cell death and suppress tumor growth, suggesting that TSN can be used as a positive synergist to help chemotherapy medication overcome drug resistance.

Targeted therapy and immunotherapy have merged as promising options for tumor treatment in recent years. However, the frequent emergence of tumor drug resistance has become one of the major obstacles to successful treatment. Increasing evidence indicates that combination therapy is the key to optimizing prescription, reducing cost, and prolonging cancer patients' survival. *KRAS* mutation is a critical genetic driver for the development of pancreatic cancer, colorectal cancer, and lung cancer. A recent study showed that pharmacologic or genetic autophagy inhibition can synergistically enhance ERK inhibitor-induced cancer cell death in KRAS-driven pancreatic ductal adenocarcinoma 59. Also, advances in immunotherapy have produced significant and effective clinical responses in some cancer patients. However, immune tolerance is often an inevitable obstacle that limits the long-term effectiveness of immunotherapy. Another study provided convincing data showing that the combination of early-stage autophagy inhibitors can turn cold to hot and inflamed tumors and improves the treatment of anti-PD-L1/PD-1 in multiple tumor models 60. In general, we can see that autophagy inhibition is an important strategy to enhance the anti-cancer effects of targeted therapy and immunotherapy. As a potent autophagy inhibitor, TSN may have the potential to become an effective drug resistance reversal agent and positive synergist in fighting against malignant tumors.

## Materials and Methods

### Antibodies and reagents

Primary antibodies: β-actin, cathepsin B, cleaved caspase-3, cytochrome C, GAPDH, LAMP1 (Cell Signaling Technology, 4970, 31718, 9664, 4280, 5174, 9091), cathepsin D (Santa Cruz Biotechnology, sc-6486), LC3B (Novus, NB100-2220), SQSTM1/p62 (Abcam, ab109012), V_0_a1 and V_1_A (Proteintech, 13828-1-AP, 17115-1-AP). Secondary antibodies: rabbit IgG (Cell Signaling Technology, 7074), goat IgG (Santa Cruz Biotechnology, sc-2354), goat anti-rabbit IgG (H+L) cross-adsorbed secondary antibody, Alexa Fluor Plus 647 (Thermo Fisher Scientific, A32733).

Bafilomycin A_1_, KCl, phospho(enol)pyruvic acid monopotassium salt, Triton X-100 (Aladdin, B101389, P112143, P119478**,** T109027), L-lactic dehydrogenase, NADPH-Na_4_, pyruvic kinase (Beijing Solarbio Science & Technology Co., Ltd., L8080, N8100, P8200), bovine serum albumin (BSA), HEPES, RIPA buffer, QuickBlock, LDH cytotoxicity assay kit (Beyotime Biotechnology, ST023, ST092, P0013C, P0013D, P0260, C0016), β-mercaptoethanol (GIBCO, 21985-023), torin1 (LC Laboratories, T-7887), sucrose (Macklin Inc, S818046), protease inhibitor cocktail (MedChemExpress, HY-K0010), 4-dimethylaminopyridine (4-DMAP), 6-biotinamidohexanoic acid, CaCl_2,_ camptothecin, EDCI, EDTA Na_2_, N,N-dimethylformamide (DMF), toosendanin (Shanghai XianDing Biological Science & Technology Co. Ltd, D001A, B-ML407, AQ461, HZ306, D001F, ER195, NK300, HN057), chloroquine, DMSO, paraformaldehyde (Sigma, C6628, D8418, 158127), ouabain octahydrate, SAR405, sodium azide, Tween 20 (Sigma-Aldrich, O3125, 5330630001, S2002, P2287), Dulbecco's Modified Eagle Medium (DMEM), fetal bovine serum (FBS), penicillin and streptomycin antibiotics, Dulbecco's Phosphate-Buffered Saline (PBS), BCA protein concentration assay kit, Hoechst 33342, Poly-D-Lysine, LysoTracker Red DND-99, DQ Red BSA, Propidium iodide (PI), Sheared Salmon Sperm DNA, trypsin (Thermo Fisher Scientific, 12100046, 10270106, 10378016, 21600010**,** 23225, H1399, L7528, A3890401, D12051, P3566, AM9680, 25200072). PVDF membrane, non-fat milk (Bio-Rad Laboratories, 1620177, 170-6404). ECL (GE Healthcare, 45-002-401).

### Cell culture

HeLa and A549 cells were cultured in Dulbecco's Modified Eagle Medium supplemented with 10% fetal bovine serum and 1% penicillin and streptomycin antibiotics in a humid incubator with 5% CO_2_ at 37 ℃.

### Western blotting analysis

After indicated concentration or time drug treatment, cells were washed with PBS and lysed in RIPA buffer (1% NP-40, 0.5% deoxycholate, 0.1% SDS) supplemented with protease inhibitor cocktail for 20 min on ice and centrifuged for 20 min at 15,000 × g at 4 ℃. A BCA protein concentration assay was performed to determine protein concentration. The cell lysate was added with SDS-PAGE loading buffer and heated at 98 ℃ for 5 min. Samples (10~30 μg) were resolved through 8~12% SDS-PAGE gels, next transferred onto the PVDF membrane, and then blocked with non-fat milk in tris buffer saline (pH 7.4) with 0.1% tween. After incubation with specific primary antibodies at 4 ℃ overnight and secondary antibodies at room temperature (25 ℃) for 1 h by gentle shaking, immunoreactivity was detected by ECL. The protein bands intensity was quantified by ImageJ.

### Autophagosome-lysosome fusion process analysis

HeLa and A549 cells stably expressing mRFP-GFP-LC3B were grown on the Poly-D-Lysine treated-cover glass slips in the 24-well culture plates. After treatment, cells were washed with PBS and fixed with 4% paraformaldehyde for 15 min, then washed with PBS. Next, cells were incubated with 0.1% Triton X-100 for 15 min, then washed with PBS and blocked with QuickBlock for 30 min. Cells were incubated with anti-LAMP1 primary antibody at 4 ℃ for 6 h and then Alexa Fluor 647-labeled secondary antibody (showed in blue) at room temperature for 1 h. Cells were imaged using the LSCM (Leica, TCS SP8, Leica Microsystems Inc., New York, US). The colocalization analysis was quantified by ImageJ.

### Lysosomal acidic environment analysis

HeLa and A549 cells were grown on the Poly-D-Lysine treated-cover glass slips in the 24-well culture plates. After treatment, cells were washed three times with serum-free culture medium, incubated with LysoTracker Red DND-99 (working solution, 50 nM) at a humid incubator with 5% CO_2_ at 37 ℃ for 1 h, washed three times with PBS, fixed with 4% paraformaldehyde for 15 min, washed three times with PBS, stained with Hoechst for 10 min, and washed three times with PBS. Images were visualized using LSCM (excitation wavelength 577 nm, emission wavelength 590 nm). The fluorescence intensity was quantified by ImageJ.

### Lysosomal proteases hydrolytic activity analysis

HeLa and A549 cells were grown on the 12-well culture plates and pre-incubated with DQ Red BSA (working solution, 10 μg/mL) at a humid incubator with 5% CO_2_ at 37 ℃ for 2 h, and then treated with 0.1% DMSO or each compound for 6 h. Images were visualized using the IncuCyte S3 live-cell analysis system (Sartorius, Germany). The fluorescence intensity was quantified by ImageJ.

### Rat liver lysosomes isolation process

Lysosomes were isolated from Sprague-Dawley rat liver with the sucrose density gradient centrifugation as described 61-63 with modifications: 8-week-old male rat (200~250 g) starved for 8 h, isolated the liver (8~10 g) and removed the gall bladder and fat tissue. Washed extensively in ice-cold 0.25 M sucrose solution (containing 10 mM HEPES, pH 7.5, 0.25 M sucrose, 1 mM EDTA-Na_2_) and cut into small pieces, homogenized in a total volume of 10 mL of the same buffer (1 mL solution per gram of liver) by the glass homogenizer. Filtered the homogenate through a 75-μm cell strainer (Beijing Solarbio Science & Technology Co., Ltd., YA0949) and split each 8 mL homogenate into 15-mL polycarbonate tubes. The ultrasonic cell breaker (SHUNMATECH, SM-150D, Nanjing, China) was used to break the cell membranes (working parameters: ultrasonic energy=87.5 W, temperature=4°C, each ultrasonic time=2 s, interval time=3 s, total ultrasonic time=15 min). The homogenate was centrifuged at 1000 g for 10 min. Collected the supernatant and split it into the 13.2 mL thin-wall polypropylene tube (Beckman-Coulter, 331372). Centrifuged at 11,000 g for 20 min in the SW 41Ti rotor (Beckman-Coulter, California, US). Removed the supernatant and resuspended the pellet in the ice-cold CaCl_2_ solution (10 mM CaCl_2_ supplemented with protease inhibitor cocktail) to make the mitochondria swell and become less dense, thus promoted their separation from lysosomes. Transferred the organelle pellet into a 50-mL polycarbonate tube, added the ice-cold CaCl_2_ solution to 50 mL, and incubated the suspension in a rotator for 30 min. Split it into four 13.2-mL thin-wall polypropylene tube and centrifuged at 20,000 g for 25 min in the SW 41Ti rotor. Removed the supernatant, added 4 mL 0.25 M sucrose solution, and resuspended the pellet (postorganellar supernatant). Constructed a step sucrose gradient in the following method: 4 mL of 0.41 g mL^-1^ (41% w/v) sucrose solution (dissolved in 10 mM HEPES buffer, pH 7.5) was overlaid with 4 mL of 0.2 g mL^-1^ (20% w/v) sucrose solution, and finally with 4 mL postorganellar supernatant and centrifuged at 112,700 g for 4 h in the SW 41Ti rotor. The band at the 20/41% sucrose solution interface was collected as the lysosomal fraction. All the steps were performed on ice or at 4 ℃. The protein concentration of the lysosomal fraction was determined by the BCA assay. The enrichment of lysosomes was confirmed by detecting LAMP1, CTSB, and cytochrome C protein levels through WB. The lysosomal fraction was mixed in PBS (pH 7.4), 40% glycerol, aliquoted, and stored at -80 ℃.

### Rat liver lysosomal V-ATPase activity assay

V-ATPase activity was measured by ATP/NADPH-coupled assay as described 34, 35 with modifications: calculated the required number of control and treatment groups, and prepared the total volume of reaction buffer with slight excess. 1 mL reaction buffer contained 10 mM HEPES-NaOH (pH 7.4), 100 μg mL^-1^ BSA, 5 mM β-mercaptoethanol, 150 mM KCl, 0.16 mM NADPH, 3 mM phosphoenol-pyruvate (PEP), 5 U pyruvate kinase (PK), 8 U lactate dehydrogenase (LDH), 1 μM ouabain (inhibits Na^+^, K^+^-ATPase), 5 mM Na azide (inhibits the mitochondrial F_0_F_1_-ATPase). 8.5 μg lysosomal fraction and the indicated concentrations of BAF or TSN were mixed with reaction buffer in a total volume of 120 μL and transferred into a 96-well culture plate (NUNC, Denmark, 167008). Incubated the reaction samples at 37 ℃ for 30 min. The reactions were started by the addition of 30 μL of 15 mM Mg-ATP, and the OD values were recorded on the FlexStation 3 Multi-Mode Microplate Reader (Molecular Devices, California, US) for 10 min, subtracted background levels from all wells. The ATP hydrolysis activity was calculated by the formula: [treatment group: initial OD value - final OD value]∕[control group: initial OD value - final OD value]. V-ATPase activity inhibition was calculated by the formula: [1 - ATP hydrolysis activity].

### Yeast V-ATPase purification

V-ATPase was purified as described 64 with modifications: *Saccharomyces cerevisiae* strain SABY31 (3× FLAG tag on Vma1p, with the *stv1* gene deleted) was grown at 30 ℃ on YPD agar (1% w/v yeast extract, 2% w/v peptone, 2% w/v D-glucose, 1.5% w/v agar) and used to inoculate two 100 mL cultures of YPD liquid medium. These cultures were grown at 30 ℃ overnight (>12 h) with shaking at 220 rpm. The cultures were then used to inoculate YPD (11 L) in a Microferm fermentor (New Brunswick Scientific Co., Inc., New Jersy, US), which was incubated for 2 d (36~48 h) with stirring at 300 rpm and aeration at 34 cubic feet per hour. 1.5 h before harvest of yeast, 1 L of 22% w/v D-glucose was added to the culture.

All cell harvest and protein purification steps were performed at 4 ℃. Cells were harvested by centrifugation at 4000 × g for 15 min. Cell pellets were resuspended in lysis buffer at 1 mL g^-1^ cell pellet (10 mM Na_2_HPO_4_ and 2 mM KH_2_PO_4_ pH 7.4, 140 mM NaCl, 3 mM KCl, 8% w/v sucrose, 2% w/v D-sorbitol, 2% w/v D-glucose, 5 mM 6-aminocaproic acid, 5 mM benzamidine, 5 mM EDTA, 0.001% w/v PMSF). Yeast was lysed in a bead beater with 0.5-mm glass beads (Bio Spec Products Inc., 11079105), cooled with ice, using 4 cycles of 1 min of bead-beating followed by 1 min of cooling. Cell debris was removed by centrifugation at 3000 × g for 10 min, and membranes were collected by centrifugation at 110,000 × g for 40 min. Cell membranes were resuspended in lysis buffer at 0.5 mL g^-1^ original cell pellet, divided into 30 to 45 mL fractions, flash-frozen in liquid nitrogen, and stored at -80 ℃.

Membranes were thawed and solubilized with 1% w/v n-dodecyl β-D-maltopyranoside (DDM). Insoluble material was removed by centrifugation at 150,000 × g for 70 min. The supernatant containing solubilized membranes was filtered with 0.45-μm filters and bound to 0.8 mL of anti-FLAG M2 agarose beads (Sigma) in a disposable plastic column. The beads were washed with 10-bed volumes of DTBS (50 mM Tris-HCl pH 7.4, 150 mM NaCl, 0.05% w/v DDM). V-ATPase was eluted with 1.5-bed volumes of DTBS buffer containing 150 μg mL^-1^ FLAG peptide and an additional 1-bed volume of DTBS. The sample was then concentrated for 15 min at 2000 × g in a VivaSpin6 concentrator to ~100 μL, and concentrations were measured by the BCA assay.

### Yeast V-ATPase activity assay

Enzyme-coupled ATPase activity assay was performed as described 65-67 with modifications: assays were performed in a 96-well culture plate with a total reaction volume of 160 μL. Purified yeast V-ATPase (0.347 μg) was added to the assay buffer (50 mM Tris-HCl pH 7.4, 3 mM MgCl_2_, 0.2 mM NADH, 3.2 U PK, 8 U LDH, 0.02% w/v DDM, and 100 μg mL^-1^ soybean asolectin). Inhibitors in DMSO were added from stock solutions, with the final concentration of DMSO in the reaction maintained at 0.1% v/v. Plates were incubated at 37 ℃, and reactions were initiated by the addition of ATP and PEP to 2 mM and 1 mM, respectively. Absorbance at 340 nm was monitored at 37°C for 10 min, and the linear regions of the curves prior to NADH depletion were used to calculate the rate of change for absorbance with time.

### Co-immunoprecipitation assay

Prepare cell lysate: grew the cells to approximately 80% confluence. After treatment, cells were washed with PBS and lysed in RIPA buffer (1% NP-40, 0.25% deoxycholate) supplemented with the protease inhibitor cocktail for 20 min on ice, and centrifuged for 20 min at 15,000 × g at 4 ℃. The BCA protein concentration assay was performed to determine protein concentration. Pre-block the magnetic beads: washed 150 μL Dynabeads Protein G (Thermo Fisher Scientific, 10004D) with 850 μL PBS by the gentle vortex. Centrifuged for 2 min at 2,000 × g at room temperature, placed the tube on the magnetic separation rack, and removed the supernatant. Resuspended the magnetic beads in a total volume of 1 mL blocking agent, containing 3% BSA, 20 μL Sheared Salmon Sperm DNA and 980 μL PBS (pH 7.4) with 0.1% tween (PBS-T), incubated on a rotator at room temperature for 1 h. Washed the magnetic beads and resuspended in 150 μL PBS-T, kept at 4°C before use. Pull down V_1_A: 2 μg IgG was mixed with 3 mg 0.1% DMSO-treated cell lysate and PBS-T in a total volume of 1 mL, 2 μg V_1_A was mixed with 3 mg 0.1% DMSO-treated or TSN-treated cell lysate and PBS-T in a total volume of 1 mL, incubated on a rotator at 4 ℃ for 6 h. Transferred each sample to a new 2-mL microcentrifuge tube, added 50 μL pre-blocked magnetic beads into each sample, and incubated on a rotator at 4 ℃ for 4 h. Centrifuged for 2 min at 2,000 × g at room temperature, transferred each sample to a new microcentrifuge tube, placed the tubes on the magnetic separation rack, removed the supernatant, added 500 μL PBS, and rotated on a rotor at room temperature for 5 min. Repeated the washing step 6 times. After the last washing step, removed as much liquid as possible and added SDS-PAGE loading buffer and heated at 98 ℃ for 5 min. 2 μg/μL 0.1% DMSO or TSN-treated cell lysate was prepared and used as the input group.

### Immunoprecipitation-coupled LC-MS/MS analysis for the detection of label-free compound-protein physical binding

Pre-block the magnetic beads: 150 μL Dynabeads Protein G was pre-blocked as mentioned above. Prepare the lysosomes lysate: lysosomal fraction (2 mg) was mixed with 200 μL RIPA buffer (1% NP-40, 0.25% deoxycholate), protease inhibitor cocktail, and PBS in a total volume of 1.5 mL and incubated on a rotator at 4 ℃ for 30 min. Prepare TSN-treated lysosomes lysate: 10 μM TSN was mixed with lysosomes lysate and incubated on a rotator at 4 ℃ for 2 h, evenly distributed into three 2-mL microcentrifuge tubes. Simultaneously, prepared a blank group with the same volume of PBS, the same concentration of TSN, but with no lysosome lysate, incubated at the same time to observe the effect of the microcentrifuge tube wall on the adhesion of the drug. Pull down V-ATPase: 5 μg IgG or V_1_A or V_0_a1, was mixed with TSN-treated lysosomes lysate and PBS-T in a total volume of 1 mL and incubated on a rotator at 4 ℃ for 6 h. Transferred each sample to a new 2-mL microcentrifuge tube, added 50 μL pre-blocked magnetic beads into each sample, and incubated on a rotator at 4 ℃ for 4 h. Centrifuged for 2 min at 2,000 × g at room temperature, transferred each sample to a new microcentrifuge tube, placed the tubes on the magnetic separation rack, removed the supernatant, added 500 μL PBS, and rotated on a rotor at room temperature for 5 min. Repeated the washing step 6 times. After the last washing step, removed as much liquid as possible, and added 100 μL elution buffer (50 mM glycine, pH 2.8), gently tapped the tube for 5 min to dissociate the magnetic bead-antibody-target protein complex. Centrifuged for 2 min at 2,000 × g at room temperature, placed the tubes on the magnetic separation rack, carefully transferred the supernatant to new 1.5-mL microcentrifuge tubes. Collected 3 individual samples and stored at -80 ℃ for the following analysis.

Establish the calibration curve for quantification of TSN concentration: prepared 1 μM stock solution of TSN (dissolved in methanol), and diluted TSN to 0.5~100 nM for the following analysis. Prepare samples for LC-MS/MS analysis: 50 μL each sample was added into 100 μL methanol, vortexed for 3 min, and incubated for 10 min at room temperature to ensure complete protein precipitation. Centrifuged for 10 min at 15,000 × g at 4 ℃ and carefully transferred 100 μL supernatant into the autosampler vial. LC-MS/MS conditions: samples were analyzed with Agilent 1200 Series HPLC (high-performance liquid chromatography) system (Santa Clara, California, US) connected to AB SCIEX 4000 QTRAP LC-MS/MS system (Framingham, Massachusetts, USA) under negative ion multiple reaction monitoring (MRM) modes. Chromatographic separation was performed on the Agilent ZORBAX SB-C18 column (100 mm × 4.6 mm, 3.5 μm) with the isogradient mobile phases (water:0.01% v/v acetonitrile=55:45) at 35 ℃ and the flow rate of 0.6 mL min^-1^. The injection volume was 20 μL; the total run time was within 7.5 min. Optimal MS parameters: ion spray voltage, -4.5 kV; curtain gas, 40 psi; collision gas set at high, capillary temperature 550 ℃ and both ion sources of gas (nitrogen) at 55 psi; declustering potential were -88 eV, entrance potential -10 eV, collision cell exit potential -12 eV, and collision energy were -30 eV. The MRM transitions were m/z 573.4/531.4 for the quantification of TSN concentration.

### Cellular thermal shift assay (CETSA)

CETSA was performed as described 68, 69 with modifications: HeLa cells were seeded in the 100-mm culture dishes to 80% confluency and treated with FBS-free medium containing 0.1% DMSO or 10 μM TSN for 1 h in a humid incubator with 5% CO_2_ at 37 ℃. After treatment, cells were washed with PBS, detached with trypsin, and collected with medium supplemented with 10% FBS. The suspension was centrifuged at 250 × g at room temperature for 3 min, washed twice with PBS, and resuspended in 1 mL PBS. Each 90 μL suspension was aliquoted into a 0.2-mL PCR tube, heated for 3 min at indicated temperature (43 to 71 ℃) in a PCR machine (SureCycler 8800, Agilent Technologies, California, US), and rapidly cooled to room temperature. Next, added 20 μL RIPA buffer (1% NP-40, 0.25% deoxycholate) into each tube, mixed, and rotated on a rotor at 4 ℃ for 20 min. Centrifuged for 20 min at 15,000 × g at 4 ℃ to eliminate the precipitated proteins. Transferred 100 μL supernatant to a new 1.5-mL microcentrifuge tube, added 25 μL SDS-PAGE loading buffer (5 ×) into each tube, and heated at 98 ℃ for 5 min, and detected the protein levels of V_1_A, V_0_a1, and LAMP1.

### Synthesis of biotin-TSN

TSN (20 mg, 2 equiv) and 6-biotinamidohexanoic acid (12.4 mg, 1 equiv) were added to a solution of EDCI (4.25 mg, 1 equiv) and 4-DMAP (5.4 mg, 1 equiv) in DMF (1 mL). The solvent was stirred at 60 ℃ overnight. The reaction was monitored by the LC-MS analysis. After that, the mixture was isolated using semipreparative HPLC on an RP C18 column (250 mm × 10 mm, 5 μm) with MeCN-H_2_O (80:20) as the mobile phase obtained Biotin-TSN (10 mg). The structure of Biotin-TSN was elucidated by its HRMS and NMR data analysis.

Biotin-TSN, white solid. HRESIMS, *m/z* 914.4103 [M + H]^+^ (calcd for C_46_H_64_N_3_O_14_S, 914.4104), 936.3928 [M + Na]^+^ (calcd for C_46_H_63_N_3_NaO_14_S, 936.3923); ^1^H NMR data (CD_3_OD, 600 MHz), *δ*_H_ 0.82 (3H, s), 1.14 (3H, s), 1.38 (3H, s), 1.95 (3H, s), 2.08 (3H, s) 2.13 (1H, dd, *J* = 13.4, 6.4 Hz), 2.41 (2H, t, *J* = 7.2 Hz), 2.66 (1H, dt, *J* = 16.0, 4.9 Hz), 3.17 (2H, dd, *J* = 7.1 Hz), 3.81 (1H, s), 4.36 (1H, d, *J* = 12.8 Hz), 4.73 (1H, s), 5.13(1H, d, *J* = 4.5 Hz), 5.34 (1H, s), 5.80 (1H, s), 6.17 (1H, s), 7.20 (1H, s), 7.40 (1H, s); ^13^C NMR data (CD_3_OD, 150 MHz), *δ*_C_ 15.8,19.5, 20.9, 21.3, 22.9, 25.5, 26.5, 26.9, 27.3, 29.4, 29.5, 29.8, 30.0, 34.8, 34.9, 36.7, 36.8, 39.3, 39.9, 40.4, 41.0, 42.6, 43.9, 46.8, 50.1, 57.0, 59.8, 61.6, 63.4, 66.2, 70.3, 70.7, 73.5, 74.6, 79.7, 96.1, 113.0, 124.2, 142.1, 143.7, 166.1, 172.1, 172.7, 173.8, 176.0, and 209.1.

### Biotin-TSN pull-down assay

Pre-block the magnetic beads: 160 μL streptavidin magnetic beads (MedChemExpress LLC, HY-K0208) was pre-blocked as mentioned above. Washed the magnetic beads and resuspended in 100 μL PBS-T, kept at 4 ℃ before use. Prepare biotin and biotin-TSN conjugated beads: 50 μL magnetic beads, 10 μM biotin or 10 μM biotin-TSN, and PBS were mixed in a total volume of 1 mL, incubated on a rotator at room temperature for 1 h. Washed the magnetic beads for 6 times and resuspended in 100 μL PBS, kept at 4 ℃ before use. Prepare the lysosomes lysate: lysosomal fraction (2 mg) was mixed with 200 μL RIPA buffer (1% NP-40, 0.25% deoxycholate), protease inhibitor cocktail, and PBS in a total volume of 1.5 mL and incubated on a rotator at 4 ℃ for 30 min. Evenly distributed into two 2-mL microcentrifuge tubes. Pull down biotin and biotin-TSN target proteins: biotin or biotin-TSN conjugated beads were mixed with lysosomes lysate and PBS-T in a total volume of 1 mL, respectively, and incubated on a rotator at 4 ℃ for 6 h. Washed the magnetic beads for 3 times (Transferred each sample to a new 2-mL microcentrifuge tube after the first washing step). Centrifuged for 2 min at 2,000 × g at room temperature, placed the tubes on the magnetic separation rack, removed as much liquid as possible, added 80 μL SDS-PAGE loading buffer (1 ×) into each sample, heated at 98 ℃ for 5 min, and detected the protein levels of V_1_A and V_0_a1.

### Cell viability assay

LDH cytotoxicity assay kit was used to detect LDH release in each treatment. Cells were seeded into the 96-well culture plate at a density of 5000 cells/well and treated the indicated concentrations of CPT for 24 h, added TSN at the last 12 h. Set another two groups, in the background control group, set no cell well with 200 μL culture medium. In the maximum release group, seeded the same density cells, and added cell lysis solution and mixed adequately at the last 23 h, incubated for another 1 h. After treatment, centrifuged the 96-well culture plate at 400 × g for 5 min, and transferred 120 μL supernatant to a new 96-well assay plate. Added 60 μL LDH reaction solution to each well and incubated the plate with gentle shaking on a horizontal shaker for 30 min. The plates were read on the FlexStation 3 Multi-Mode Microplate Reader at 490 nm. The cytotoxicity was calculated by the formula: [experimental OD value - background control OD value]∕[maximum release OD value - background control OD value]. The cell viability was normalized to the control group.

### Cell death analysis

Propidium iodide (PI) staining was used to detect cell death in each treatment. Cells were seeded into the 96-well culture plate at a density of 5000 cells/well and treated the indicated concentrations of CPT for 24 h, added TSN at the last 12 h. After treatment, centrifuged the 96-well culture plate at 400 × g for 5 min. Cells were incubated with PI (working solution, 3 μM) at a humid incubator with 5% CO_2_ at 37 ℃ for 30 min, washed three times with PBS. Images were visualized using the IncuCyte S3 live-cell analysis system (red fluorescence channel).

### Colony formation assay

Cells were seeded into the 6-well culture plate at a density of 1000 cells/well and treated with each compound for 7~10 d (gently replaced the culture medium every two days). After treatment, cells were gently washed with PBS, fixed by 4% paraformaldehyde for 15 min, gently washed with PBS, stained with 2 mg mL^-1^ (0.2% w/v) crystal violet for 5 min, gently washed with PBS, dried in the air, and photographed. The colony numbers (> 50 cells) were counted.

### Tumor xenograft experiment

The animal research ethics application was approved by the University of Macau (Approval No. UMARE-003-2018). 40 female nude mice at 5 weeks of age were purchased at the Faculty of Health Sciences from the University of Macau. For each nude mouse, 1 × 10^6^ HeLa cells stably expressing mRFP-GFP-LC3 were suspended in 100 μL serum-free culture medium and inoculated into the left axilla. After 7 d, the tumors in 34 mice reached 100~150 mm^3^ in size. These 34 mice were randomly allocated into 5 groups and received the intraperitoneal injection of 20% intralipid (FRESENIUS KABI SSPC, Intralipid 20% Injection 250 mL), TSN (0.5 or 1 mg/kg), CPT (2 mg/kg), and CPT (2 mg/kg) plus TSN (0.5 mg/kg) every 2 days for 3 weeks. TSN or CPT was dissolved in 20% intralipid. Mice were sacrificed 21 d after treatment. Tumors were isolated and tested by WB or immunofluorescence analysis.

### Histology and immunofluorescence

Part of the tumors was fixed in 4% paraformaldehyde for 1 d, followed by the dehydration process in 0.2 g mL^-1^ (20% w/v) and 0.3 g mL^-1^ (30% w/v) sucrose for 1 d, respectively, and embedded with optimum cutting temperature (O.C.T.) (SAKURA, 4583) at -80 ℃. 6 μm tumor pieces were cut by the freezing microtome (Leica CM1950, Buffalo, USA), then incubated at 37 ℃ for 1 h, washed three times with PBS. Next, tumor tissues were blocked with QuickBlock for 30 min, incubated with anti-cleaved caspase-3 primary antibody at 4 ℃ overnight, and then with Alexa Fluor 647-labeled secondary antibody at room temperature for 1 h. The cell nuclei were stained with Hoechst. Tumor tissues were imaged using the LSCM. Livers, kidneys, and spleens from each group were fixed in 4% paraformaldehyde, embedded in paraffin and cut into 5 μm pieces, and then performed H&E staining. The images were captured using the microscope.

### Statistical analysis

All experimental data were acquired from at least 3 individual samples. Results were expressed as means ± standard deviation (SD) using Prism 8.0 (GraphPad Software, California, USA). The differences between groups were analyzed by one-way ANOVA with Tukey's post-hoc test. Differences at *P*<0.05 were considered statistically significant (^*^P< 0.05, ^**^P<0.01, ^***^P<0.001; ^#^*P*<0.05, ^##^*P*<0.01, ^###^*P*<0.001 [*vs*. Control group]).

## Supplementary Material

Supplementary figures.

## Abbreviations

ATG: autophagy-related
ATP: adenosine triphosphate
BAF: bafilomycin A_1_
CPT: camptothecin
CQ: chloroquine
CTSB: cathepsin B
CTSD: cathepsin D
GFP: green fluorescent protein
i.p.: intraperitoneal injection
LAMP1: lysosomal-associated membrane protein 1
LC-MS: liquid chromatography-mass spectrometry
LSCM: laser scanning confocal microscopy
MAP1LC3/LC3: microtubule-associated protein 1 light chain 3
MS: mass spectrometry
mRFP: monomeric red fluorescent protein
PI: propidium iodide
SQSTM1: sequestosome 1
TSN: toosendanin
V-ATPase: vacuolar-type H^+^-translocating ATPase
WB: western blotting
