# Supplementary material for: Toosendanin, a novel potent vacuolar-type H+-translocating ATPase inhibitor, sensitizes cancer cells to chemotherapy by blocking protective autophagy
Source: Int J Biol Sci. 2022 Mar 28;18(7):2684–702. doi: 10.7150/ijbs.71041 (PMC9066121; doi:10.7150/ijbs.71041)
Supplement: Supplementary file 1 — Supplementary figures. [file ijbsv18p2684s1.pdf]

Supplementary materials

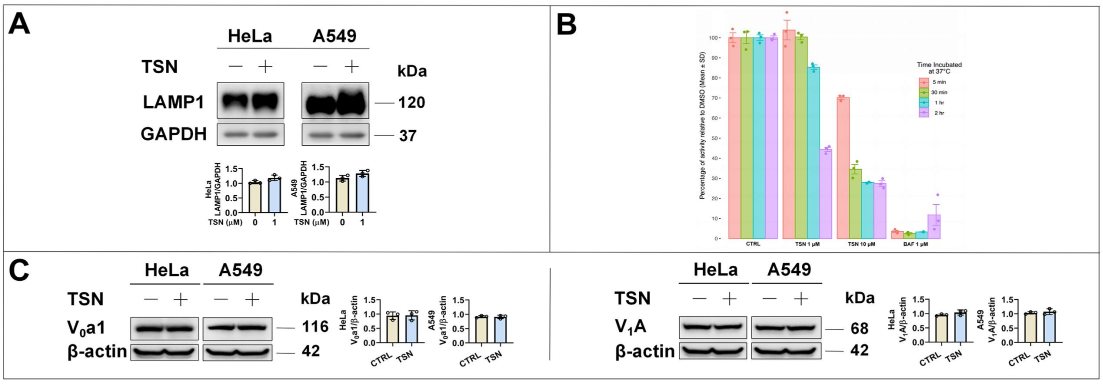

Figure S1. TSN inhibits V-ATPase activity without affecting the lysosome numbers and protein levels of V-ATPase subunits. (A) WB analysis of LAMP1 protein level in HeLa and A549 cells treated with 0.1% DMSO or TSN 1 μM for 24 h (*n*=3). (B) Purified yeast V-ATPase activity assay in BAF and TSN treatment groups (*n*=3). (C) WB analysis of V_0a1 and V_1A protein levels in HeLa and A549 cells treated with 0.1% DMSO or TSN 100 nM for 12 h (*n*=3).

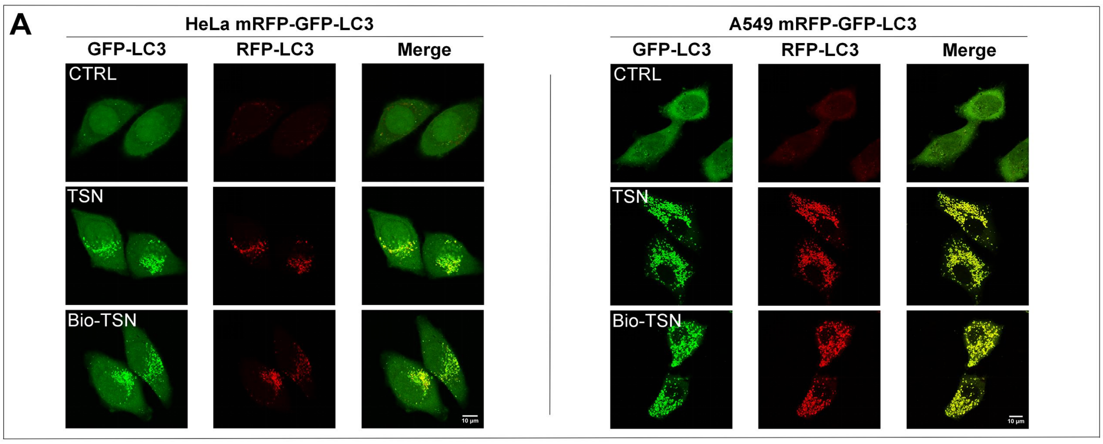

Figure S2. Bio-TSN displayed similar activity to TSN in inhibiting autophagy. (A) HeLa and A549 cells stably expressing mRFP-GFP-LC3 were treated with TSN or bio-TSN 1 μM for 12 h, and LSCM was applied to capture the fluorescent images (scale bar = 10 μm).

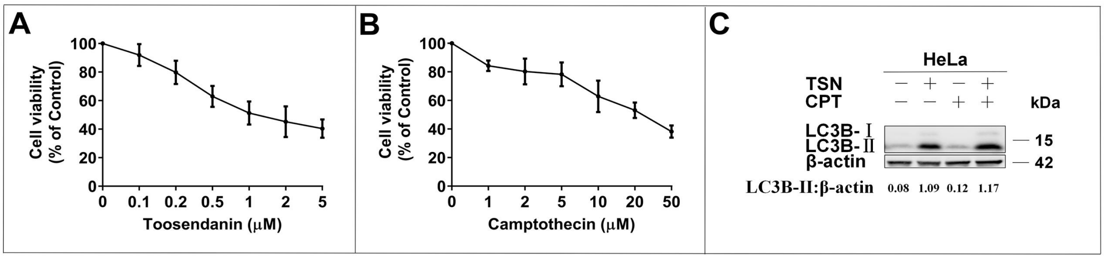

Figure S3. Cell viability and autophagy modulation of TSN and CPT. (A)-(B) HeLa cells were treated with TSN and CPT for 24 h, and MTT assay was performed to assess the cell viability (*n*=3). (C) WB analysis of LC3B-II protein level in HeLa cells treated with CPT 10 nM in the absence or presence of TSN 1 nM for 7 d.

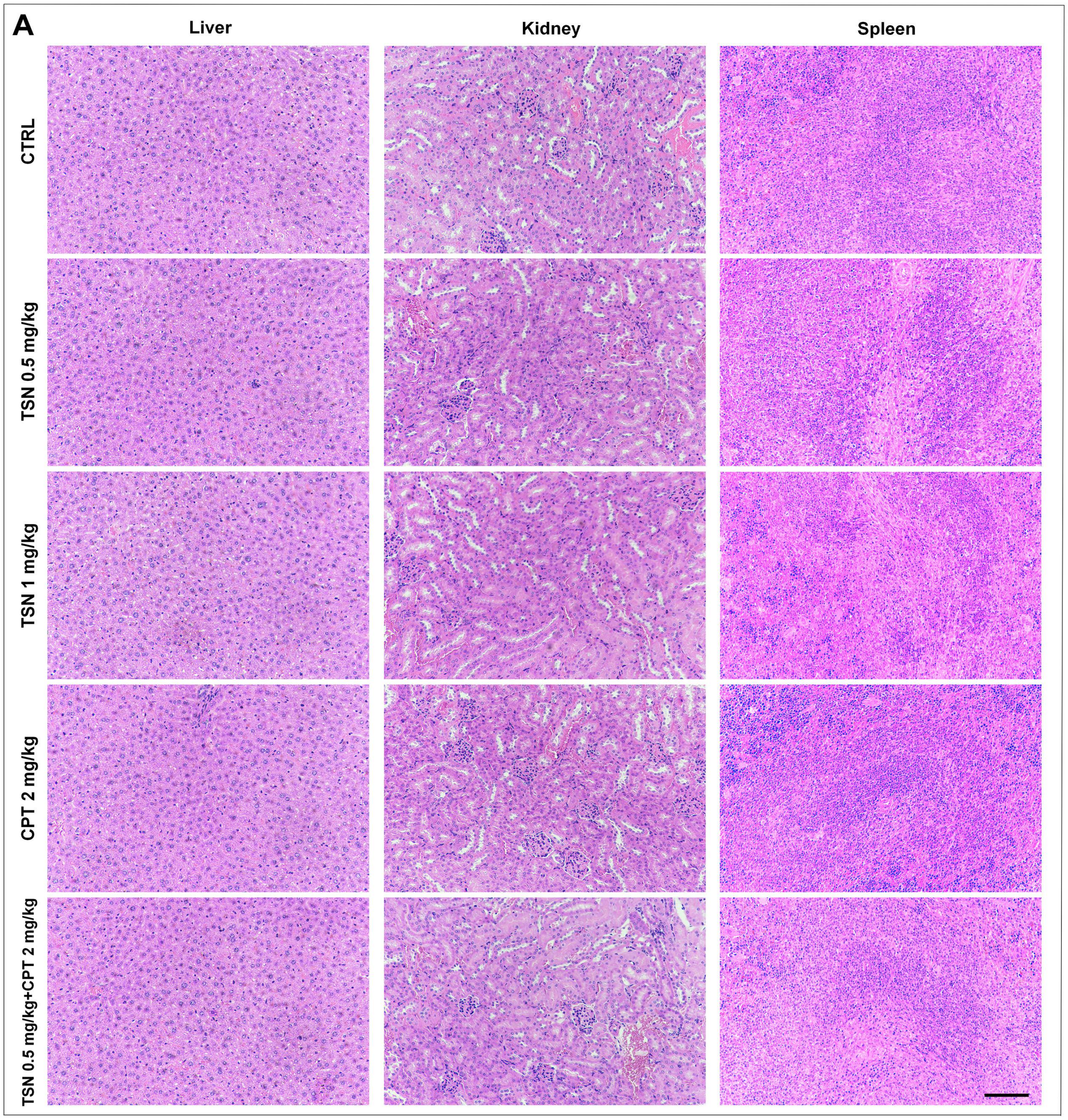

Figure S4. Histological analysis showed that administration of TSN and CPT did not cause the obvious damage and morphology change of the major organs from sacrificed nude mice. (A) Representative images showed the H&E staining of livers, kidneys, and spleens in each group, and microscope was applied to capture the images (scale bar = 100 μm).
